# Antioxidant Activities, Metabolic Profiling, Proximate Analysis, Mineral Nutrient Composition of *Salvadora persica* Fruit Unravel a Potential Functional Food and a Natural Source of Pharmaceuticals

**DOI:** 10.3389/fphar.2017.00061

**Published:** 2017-02-14

**Authors:** Asha Kumari, Asish K. Parida, Jaykumar Rangani, Ashok Panda

**Affiliations:** ^1^Division of Plant Omics, Council of Scientific and Industrial Research – Central Salt and Marine Chemicals Research InstituteBhavnagar, India; ^2^Academy of Scientific and Innovative Research, Council of Scientific and Industrial Research – Central Salt and Marine Chemicals Research InstituteBhavnagar, India

**Keywords:** antioxidant, cysteine, glucopyranose, metabolites, nutraceuticals, pharmaceuticals polyphenol, proximate composition, *Salvadora persica*

## Abstract

*Salvadora persica* is a medicinally important plant mainly used in oral hygiene. However, little attention has been given towards the nutritional prominence of this plant. This study encloses the proximate and mineral nutrient contents, amino acid composition, metabolite profiling and antioxidant potential of *S. persica* fruit. The ripen fruit contained substantial amount of sugars, mineral nutrients, carotenoids, polyphenols and flavonoids. The metabolic profiling of the fruit extract by GC-MS revealed a total of 22 metabolites comprising of sugars, sugar alcohols, organic acids, organic base, and aromatic silica compound. The identified metabolites have been previously reported to have potential antioxidant, antimicrobial, anti-hyperglycemic, and antitumor properties. The GC-MS analysis indicated high glucose and glucopyranose (247.62 and 42.90 mg g^-1^ FW respectively) contents in fruit of *S. persica*. The fruit extract demonstrated a significantly higher antioxidant and ROS scavenging properties along with high contents of mineral nutrients and essential amino acids. HPLC analysis revealed presence of essential and non-essential amino acid required for healthy body metabolism. The cysteine was found to be in highest amount (733.69 mg 100 g^-1^ DW) among all amino acids quantified. Specifically, compared to similar medicinal plants, previously reported as a source of non-conventional food and with some of the commercially important fruits, *S. persica* fruit appears to be a potential source of essential mineral nutrients, amino acids, vitamins (ascorbic acid and carotenoid) and pharmaceutically important metabolites contributing towards fulfilling the recommended daily requirement of these for a healthy human being. This is the first report establishing importance of *S. persica* fruit as nutraceuticals. The data presented here proposed that fruit of *S. persica* may be used as functional food or reinvigorating ingredient for processed food to reduce deficiency of nutrients among the vulnerable population group. The phytochemicals identified from *S. persica* fruit may be used as natural source for pharmaceutical preparations.

## Introduction

Fruits are the crucial part of human diet. They provide health benefits and helps in preventing illness. Fruits contain variety of nutrients including vitamins, minerals, bioactive compounds, and phytochemicals, especially antioxidants which help in reducing risk of chronic diseases. Fruits are naturally rich in fiber, potassium, iron, vitamin C and low in sodium, calories and fat (Ene-Obong et al., 2016). Other than the conventional source of foods which are known as the staple foods, some foods are taken less frequently and/or on certain occasions and are called as non-conventional foods e.g., wild fruits (Slavin and Lloyd, 2012). Phytochemical and nutritional composition of these common conventional food sources have been studied extensively and their nutritive value is well established (Rathore, 2009). Despite the fact that non-conventional food plants are widely consumed and are likely to be nutritious (Abitogun, 2010; Olayiwola et al., 2013), sufficient information on the phytochemical composition of the wild fruits are still missing. There are practically very less information available on the nutritive value of the wild non-conventional fruit plants which significantly contribute to the nutrient uptake of the local population (Duhan et al., 1992). Thus, exploring and understanding the phytochemical composition and antioxidant potential of these non-conventional plants may encourage utilization of these plants as a source of antioxidants and their acceptability for nutraceutical and pharmaceutical purposes.

**FIGURE 1:**
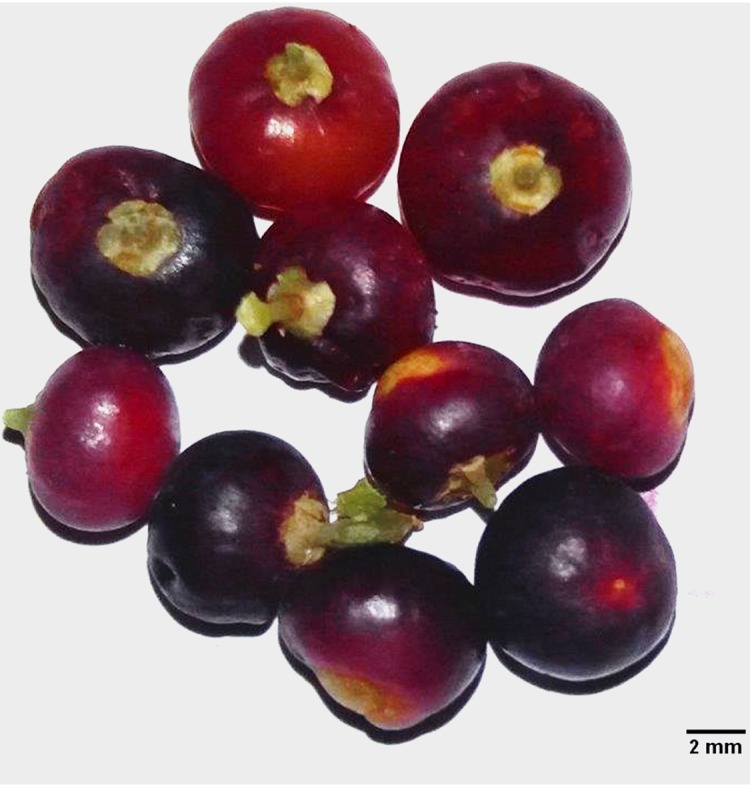
**Ripen fruit of *Salvadora persica***.

*Salvadora persica* is a medicinally important halophytic plant of family Salvadoraceae with great ethnobotanical importance. It is widely used for oral hygiene and other medicinal properties. The antioxidant and nutraceutical importance of the leaf extracts of *S. persica* have been reported in our previous study (Kumari and Parida, 2016). Fruits of *S. persica* are edible having an aromatic, sweet and peppery taste and are eaten raw, dried or cooked. Fruits are drupes with persistent calyx and corolla. They are fleshy, globose, single seeded, smooth, 5–10 mm in diameter and spherical in shape (**Figure 1**). The fruits are pink to scarlet in color when mature (Sher et al., 2010). A fermented beverage having stomachic, de-obstruent, lithontriptic, and carminative properties is made from the fruits. The fruits are also used in rheumatism and biliousness and believed to have good effect on snake bite. Besides this they are also considered as diuretic, purgative and liver tonic (Akhtar et al., 2011). Very less research information are available on this highly valuable fruit. It is, therefore, the objective of this study is to determine the nutrient, bioactive compositions and antioxidant properties of this underexploited fruit of *S. persica*. The present work will provide useful information on the prospective of *S. persica* fruit as natural source for antioxidants, essential amino acids, mineral nutrients, proteins, and other metabolites. The outcomes emerging from the current study will evaluate the potential use of *S. persica* fruit in nutraceutical and pharmaceutical formulations as dietary supplements for human. Development of biologically beneficial products from the underutilized *S. persica* fruit will have dual benefits by way of health products and their commercial cultivation in salt affected coastal lands. Besides this, its farming in coastal habitats will result in carbon-budgeting and carbon sequestration helping to resolve environmental problems like climate change.

## Materials and Methods

### Chemicals and Reagents

All the chemicals and solvents used in the experiments were of HPLC and analytical grades.

### Samples

The fruits of *Salvadora persica* were obtained from experimental salt farm area of CSIR-CSMCRI, Bhavnagar, Gujarat, India (latitude 21° 47.3060 N and longitude 72° 7.4170 E). The fruits were collected from at least five different randomly selected plants of *S. persica.* Fruits that were mature, firm and free from any damage were sorted and stored at –80°C until extraction and estimation of various parameters.

### Nutrient Determination

#### Proximate Analysis

Standard protocols of AOAC (2006) were followed for the estimation of proximate composition. For the determination of moisture content, the samples were dried at 100 ± 2°C for obtaining a constant weight (AOAC, 2006). The ash content of the sample symbolized the inorganic residue after burning of the organic matter. The ash value of the sample was determined using standard AOAC (2006) methods. Briefly, 10 g of fruit pulp was placed in pre-weighed and preheated porcelain crucible and incinerated at 550 ± 50°C in a furnace. The fat content was determined by extracting the fruit pulp with petroleum ether for 12 h with shaking. The extract was collected and reduced to half by evaporation and then dried at 105°C in an oven until a constant weight was obtained. The weight obtained after drying was divided by the weight of the sample for estimation of the fat content. For estimation of the crude fiber content, the dried sample (1 g) was extracted three times with 10ml of petroleum ether. The pellet was air dried and then added 3 mL of H_2_SO_4_ (0.1275 M) followed by the addition of 17 ml of hot H_2_SO_4_ (0.1275 M). The solution was filtered by Buchner funnel after refluxing for 30 min. The insoluble residue was washed with hot water to remove the acid. Then, the residue was taken in a flask and then, 3 mL of 0.313 M NaOH was added to the residue followed by addition of hot NaOH (17 mL, 0.313 M). The mixture was shaken for 30 minutes and filtered. Then the residue was washed with 1% HCl followed by washing with boiling water until no residual acid was present in the final filtrate. Finally, ethanol and ether were used to wash the residue before drying at 100°C in an oven and after drying the weight of the residue was recorded to determine the crude fiber content. The fiber content was calculated by dividing the loss in weight of the sample by the weight of the sample and expressed as g per 100 DW. For protein estimation approximately 500 mg of fruit pulp was homogenized with pre chilled Tris buffer (50 mM, pH 8.5) using 5% PVPP. The extract was centrifuged at 4°C for 10 min with a speed of 15000 × *g*. The pellet was re-extracted with Tris buffer and the supernatants were combined for the estimation of protein content by Lowery method (Lowry et al., 1951). After measuring the absorbance at 660nm, the calculation for protein content was carried out by plotting a standard curve of Bovine serum albumin (0.1–1 mg/ml). The carbohydrate content was calculated by subtracting the sum of protein, fat, moisture and ash content from 100 (carbohydrate = 100-protein-fat-moisture-ash). For calculating the energy content Atwater factor was used (9 for total fat and 4 for carbohydrate and protein each).

#### Determination of Mineral Ion Content

Standard protocol of Pequerul et al. (1993) was followed for the determination of mineral ion contents in the fruit of *S. persica.* The fruits were dried for 48 h in an oven at 70°C. After drying, samples (0.5 g) were homogenized and kept in a volumetric flask of 25 mL capacity. Afterwards, the samples were heated for digestion after the addition of 10 mL acid mixture comprising of HClO_4_ and HNO_3_ in the ratio 4:9. Completion of digestion was marked by appearance of colorless liquid. The volume of contents in the flask was further reduced to 3–5 mL by further evaporation. After cooling, the volume was made up to 25 mL by deionized water and filtered through Whatman No. 1 filter paper. The contents of various ions in the fruit were determined by ICP-AAS (Optima 2000DV, Perkin Elmer, USA).

#### Vitamins

##### Estimation of Carotenoids

For the estimation of carotenoids, approximately 0.5 g of fruit pulp was homogenized with 1 mL of 100% *N*, *N*-dimethylformamide (DMF) in a pestle and mortar at 4°C under dark condition. Then, the homogenate was centrifuged at 15,000 × *g* for 15 min. The absorbance of the supernatant was recorded at 664.5, 647, and 461 nm by means of a microplate reader (Epoch^TM^, BioTek, USA). The equation of Chamovitz et al. (1993) was used for the estimation of carotenoids content.

##### Ascorbic Acid Estimation

A modified protocol of Kampfenkel et al. (1995) was used for the estimation of ascorbic acid. Approximately (0.5 g) fruit pulp was homogenized into a fine powder using liquid nitrogen and extracted in 1 mL of 6% TCA. The extract was centrifuged for 15 min with a speed of 10,000 × *g* and the supernatant was taken for determination of ascorbic acid. Fifty microliter aliquot of the supernatant was added to the tube containing 150 μL of 0.2 M phosphate buffer (pH 7.4), and added 50 μL distilled water, 250 μL TCA (10%), 100 μL of 3% FeCl_3_, 200 μL of H_3_PO_4_ (43%), 200 μL of 2, 2′-dipyridyl [4% (w/v) in 70% ethanol] and 100 μL of 3% (w/v) FeCl_3_. The mixture was vortexed and incubated at 42°C for 40 min before taking absorbance at 525 nm in a microplate reader (Epoch^TM^, BioTek, USA) using TCA (6%) as reference. The amount of ascorbic acid was calculated using standard curve of L-ascorbic acid (5–50 μg).

### Bioactive Compound Analysis

#### Estimation of Total Polyphenol

The total polyphenol content was determined spectroscopically following the protocol of Ene-Obong et al. (2016) using Folin–Ciocalteu reagent. Polyphenol was extracted from 0.5 g pulp with 1 mL of 100% methanol and centrifuged at 7000 × *g* for 10 min with re-extraction of the pellet twice for analysis. Hundred microliter aliquot of sample prepared was mixed with 1.150 mL milli Q water and 250 μL of Folin-Ciocalteau’s reagent (1 N) and vortexed. After 3 min incubation 1 mL of 20% Na_2_CO_3_ was added prior to boiling for 1 min in a water bath. The solution was cooled and diluted two times for recording absorbance at 650 nm in a microplate reader (Epoch^TM^, BioTek, USA). A Standard curve plotted using Gallic acid (Sigma) (10–100 μg) was used for the calculation of phenol concentration in the unknown samples.

#### Determination of Total Flavonoids

The spectroscopic determination of total flavonoid in the crude extract was carried out following the method earlier described by Chang et al. (2002). In brief, 150 μL of the crude extract was incubated at room temperature for 5 min. after mixing with 1.7 mL of 30% methanol, 750 μL of 0.5 M NaNO_2_ solution and 75 μL of 0.3 M AlCl_3_. With an addition of 500 μL of NaOH (1 M), absorbance was recorded at 415 nm. A calibration curve of quercetin (25–150 μg) was plotted for the calculation of total flavonoid content.

### Evaluation of Antioxidant Activity

#### Crude Extract Preparation for the Determination of Antioxidant Activity

*Salvadora persica* fruit pulp (5 g) was homogenized to a fine powder with liquid nitrogen and extracted in 1 mL methanol (100%). The pellet was re-extracted twice with 500 μL methanol; the supernatants were combined and stored at –20°C for further analysis.

#### Evaluation of DPPH Radical Scavenging Activity of *S. persica* Fruit

Free radical scavenging activity of *S. persica* fruit extract was evaluated using 2, 2-diphenyl-1-picrylhydrazyl (DPPH) radical scavenging method as described earlier by Kumari and Parida (2016). The methanolic extract (100 μL) was added to 900 μL of freshly prepared DPPH methanol solution (0.1 mM) and incubated in dark for half an hour at room temperature. After incubation, the absorbance was noted at 517 nm using a microplate reader (Epoch^TM^, BioTek, USA) for estimating the free radical scavenging activity. The DPPH radical scavenging (%) was plotted against various concentration of the sample for the calculation of inhibition concentration (IC_50_). The concentration of the methanolic extract required for 50 % scavenging of DPPH radicals is termed as the IC_50_ of the extract.

#### Determination of Total Antioxidant Activity of *S. persica* Fruit

ABTS radical cation decolorization assay was used for determining the total antioxidant activity of the *S. persica* fruit, according to the method earlier described by Kumari and Parida (2016). ABTS^∙+^ radical scavenging activity (%) was plotted against various concentration of the sample for the calculation of inhibition concentration (IC_50_).

#### Determination of Antioxidant Activity by Phosphomolybdenum Complex Assay

Spectrophotometric determination of the antioxidant activity of *S. persica* fruit was evaluated by the formation of a phosphomolybdenum complex as reported earlier by Kumari and Parida (2016). One milliliter of reagent containing 28 mM sodium phosphate, 0.6 M sulfuric acid, and 4 mM ammonium molybdate was mixed with 50 μL of sample solutions and incubated for 90 min in a water bath at 95°C. After cooling the reaction mixture to room temperature, the absorbance was recorded at 820 nm against a blank. The absorbance of the sample was proportional to the antioxidant activity of the sample and it was expressed as the scavenging activity of the sample.

#### Determination of Antioxidant Activity by Superoxide Anion Scavenging Activity

The superoxide anion radical scavenging assay was performed following the method earlier described by Saeed et al. (2012). Briefly, 1 mL sample of various concentration (20–100 μg/mL) was mixed with reaction mixture containing 0.5 mL phosphate buffer (50 mM, pH 7.6), 0.3 mL of 50 mM riboflavin, 0.1 mL of 0.5 mM NBT, and 0.25 mL PMS (20 mM). The reaction mixture was illuminated under a fluorescent lamp to start the reaction. The sample was incubated for 20 min before measuring the absorbance at 560 nm. The scavenging activity of the extract was calculated according to the equation stated by Saeed et al. (2012).

#### Determination of Antioxidant Activity by Hydrogen Peroxide Scavenging Activity

The antioxidant activity of the crude extract of the fruit was measured by the hydrogen peroxide scavenging activity following the protocol described earlier by Kumari and Parida (2016). Hydrogen peroxide scavenging activity (%) was plotted against various concentrations of methanolic extract for the calculation of IC_50._

#### Estimation of Reducing Power

The reducing power of the fruit extract was estimated by monitoring the reduction of Fe^3+^ to Fe^2+^ producing Perl’s Prussian blue color at 700 nm (Saeed et al., 2012). Microplate reader (Epoch^TM^, BioTek, USA) was used for recording the absorbance of the reaction mixture. Higher reducing power was indicated by higher absorbance at 700 nm.

### Metabolite Profiling by GC-MS Analysis

Standard metabolite data reporting protocol was used for the extraction of the samples for the metabolic profiling (Fernie et al., 2011). The samples were extracted following the method described by Lisec et al. (2006). Hundred micrgram of deseeded fruit pulp was weighed and homogenized into fine powder using a chilled mortar and pestle using liquid nitrogen. The metabolites extraction was carried out by adding 1.4 mL of pre-chilled methanol and with subsequent addition of internal standard (100 μL of ribitol, 1 mg/mL). The mixture was incubated at 70°C for 10 min. After incubation the mixture was cooled and centrifuged at room temperature (11000 × *g*) for 10 min. Then HPLC grade chloroform (750 μL) and water (1.4 mL) was added to the supernatant. The resulting solution was mixed properly and centrifuged for 15 minutes at 22,000 × *g* at room temperature. Hundred and fifty microliter of aqueous layer was taken and vacuum dried.

For derivatization, the residues collected after vacuum drying, were re-dissolved in 40 μL of methoxylamine hydrochloride in pyridine (20 mg/mL) and derivatized at 37°C for 2 h. Then it was mixed with 70 μL of *O*-Bis(trimethylsilyl)trifluoroacetamide(BSTFA) and incubated at 37°C for 30 min. The GC column was then injected with 2 μL of sample. The GC/MS system (GCMS-QP2010, Shimadzu, Kyoto, Japan) was equipped with an auto-sampler. The ion source was tuned to 250°C and the transfer line was set as 300°C at a rate of 14.5°C s^-1^. The scanning range for the mass spectra was 70–700 mass-to-charge ratios and it was recorded at a rate of eight scans per second. The compounds were identified based on mass fragmentation spectra and relative retention time of those standards and NIST 2014 and WILLEY 2014 libraries. The quantification of the metabolites was carried out using ribitol as an internal standard.

### Amino Acid Profiling

The amino acid profiling of *S. perica* fruit was carried out according to the method described by Kwanyuen and Burton (2010). Fruit pulp (10 mg) dried and powdered was hydrolyzed in a hot-air oven with 500 μl HCl (6 N) for 24 h in a glass vessel at 110°C and the hydrolyzed sample was dried in a vacuum descicator. After hydrolysis, the samples were neutralized by adding 500 μl of a reaction mixture containing ethanol-water–TEA (v/v, 2:2:1) along with an amino acid standard (10 μl, AAS18, Sigma, USA) to the vessels containing hydrolyzed samples. The mixtures were vortexed and vacuum-dried. For derivatization, added 500 μl of derivatization mixture containing TEA–water–PITC-ethanol in the ratio1:1:1:7(v/v) and vortexed thoroughly. After an incubation of 20 min at room temperature, the reaction mixture was vacuum-dried and re-dissolved in 400 μl of 5 mM Na_2_HPO_4_ buffer (pH 7.4) containing 5% (v/v) acetonitrile. HPLC system (Waters Alliance model, 2695-seperation module, 2996-photodiode array detector with an auto-sampler, USA) was used for the analysis of the amino acid composition after filtering the sample with 0.22 μm syringe filter. The separation of the amino acid was carried out by a gradient resulted from mixing of eluent A and eluent B with a flow rate of 1 ml min^-1^ throughout. The eluent A contained 0.05% TEA, 150 mM CH_3_COONa.3H_2_O and acetonitrile (6%) having pH 6.4, however, eluent B has a composition of water and acetonitrile in the ratio 4:6 (v/v). The amino acids eluted were recorded at 254 nm. The amino acid composition was calculated from the relative proportion of the peak area and expressed on dry weight basis.

### Statistical Analysis

The results of all the experiments were stated as mean ± standard deviation (S.D., *n* = 5) and 5 different plants were selected for experimental sampling.

## Results and Discussion

### Energy and Proximate Compositions

The proximate composition of edible part of *S. persica* fruit are presented in **Table 1**. All the nutrient and proximate compositions were assessed on dry weight basis. The edible part was 73.83 ± 5.08% of the whole ripen fruit (**Figure 1**). The ripe fruit was sufficiently dehydrated containing an average moisture content of 70.00 ± 0.97 g 100 g^-1^ FW (**Table 1**). The proximate analysis showed that the fruit contained comparable moisture content to that reported for *Salvadora oleoides* (Duhan et al., 1992) but slightly higher as compared to that reported for *Durio zibethinus* (58–69 g 100 g^-1^ FW) (Charoenkiatkul et al., 2016). The protein content of the fruit (5.92 ± 0.66 g 100 g^-1^ DW) was at par with that reported for *Syzygium cumini* (Raza et al., 2015) but was found be comparable to that of *Durio zibethinus* (Charoenkiatkul et al., 2016) and was higher than *Cola parchycarpa* (Ene-Obong et al., 2016). The carbohydrate content of the *S. persica* fruit (73.66 ± 2.17 g 100 g^-1^ DW) was in comparison to the high carbohydrate containing durian variety *Durio zibethinus* and was found to higher than that reported for leguminous plants *Bracystegia eurycoma* and *Pipper guineense* (Bolanle et al., 2014) and *Cola parchycarpa* (Ene-Obong et al., 2016). The carbohydrate content was also in comparison with some conventional carbohydrate sources like cereals (72-90 g 100 g^-1^ DW) (Adewusi et al., 1995), and thus *S. persica* fruit can serve as a potential source of carbohydrate. The lipid and dietary fiber content of the fruit was also higher than the *Durio zibethinus, Bracystegia eurycoma, Pipper guineense*, and *Cola parchycarpa* signifying that the fruit is rich in essential nutrients and can be utilized for supplementing the human diet for better health. The relatively high energy content of the fruit 141.35 kcal 100 g^-1^ DW could fulfill the daily calorie intake of the people. The results presented here demonstrate that the fruit of *S. persica* could be an important source of essential nutrients and energy that could be utilized for supplementing and enhancing the diet and health of humans.

**Table 1:** Proximate composition, vitamins, and sugars content of *Salvadora persica* fruit.

Parameters	Minimum value	Maximum value	Average^a^
Edible portion (%)	66.73	86.60	73.83 ± 5.08
Moisture (g 100 g^-1^ FW)	68.86	71.56	70.00 ± 0.97
**Proximate composition (100 g^-1^ DW)**			
Energy (kcal)	126.81	150.54	141.35 ± 10.69
Protein (g)	4.88	6.70	5.92 ± 0.66
Carbohydrate (g)	70.55	75.99	73.66 ± 2.17
Lipid (g)	8.87	13.43	11.45 ± 1.90
Ash (g)	8.46	10.09	9.37 ± 0.67
Fiber (g)	8.10	12.64	10.44 ± 1.93
**Vitamins (100 g^-1^ DW)**			
Ascorbic acid (mg)	50.23	80.76	67.99 ± 15.86
Carotenoid (μg)	9.03	13.98	11.371 ± 2.48
**Antioxidants (100 g^-1^ DW)**			
Total polyphenols (mg)	373.23	700.86	539.61 ± 120.38
Flavonoids (mg)	288.05	471.98	317.48 ± 77.59

^a^*The values are mean ± SD of five independent replicates*.

### Vitamins

The ascorbic acid (vitamin C) has the high antioxidant activity and it helps in maintaining the cellular membrane integrity. Besides this, vitamin C has also been reported to prevent formation of cancer causing N-nitroso compounds from nitrites and nitrates present in water and meat (Kaur and Kapoor, 2001). A considerably high amount of ascorbic acid was found in *S. persica* fruit (67.99 ± 15.86 mg 100 g^-1^ DW). The vitamin C content was at par with that reported for *Ziziphus mauritiana* (76 mg 100 g^-1^ DW) and was found higher than some important non-conventional fruits like *Feronia limonia, Capparis decidua*, and *Balanites aegyptiaca* (Duhan et al., 1992). As compared to other species of *Salvadora* like *Salvadora oleoides* found in the arid and semi-arid region of India, the vitamin C content was much higher indicating that a high amount of vitamin C in this plant food makes it, an important source of vitamin C in the diet of people in arid and semi-arid areas.

Carotenoids are a family of fat soluble plant pigment considered as a good source of antioxidant. Besides its esthetic role of providing color to the fruit they are also considered beneficial in reducing risk of diseases like cancer, eye and heart diseases (Johnson, 2002). The carotenoid content (11.371 ± 2.48 μg 100 g^-1^ DW) in fruits of *S. persica* was at par with the carotenoid content reported for some underutilized fruits of *Garcinia prainiana*, *Durio kutejensis*, *Baccaurea reticulate*, and *Baccaurea macrocarpa* (Khoo et al., 2008). In numerous studies, however, it was proven that supplementation of carotenoids through fruits and vegetables is more significant in reducing disease risks and bringing about the health benefits of carotenoids (Fraser and Bramley, 2004; Voutilainen et al., 2006). Therefore, *S. persica* fruit seems a promising choice for increasing the dietary intake of carotenoids.

### Bioactive Compounds

Plants are the rich source of phenolic substances of different origins and functions. Most of them are biologically active compounds of plant origin having anti-cancerous, antiviral, antibacterial properties (Manach et al., 2004). However, very less reports on the phenolic contents of non-traditional fruit plants and their antioxidant activities are available (Stratil et al., 2006). The total amount of phenolics in the fruit extract was estimated to be 539.61 ± 120.38 mg 100 g^-1^ DW (**Table 1**), which was in comparison with wild fruit of *Saba senegalensis,* *Sclerocarya birrea*, and *Diospyros mespiliformis* but was higher than *Diospyros mespiliformis*, *Ficus sycomorus*, *Lannea microcarpa*, and *Parkia biglobosa*, however, the value was lower than that reported wild medicinal plants, *Ziziphus mauritiana* and *Tamarindus indica* (Lamien-Meda et al., 2008). Similarly, the total phenolic content was at par with that reported for blueberries (670.9 mg 100 g^-1^ DW) and sour cherry (429.5 mg 100 g^-1^ DW) and was higher than green and red pepper (Marinova et al., 2005).

Flavonoids are class of polyphenolic compounds that constitute the major antioxidants in fruits and possess beneficial effect on human health. Although, flavonoids are generally considered as non-nutrients, they are important component for human diet due to their high antioxidant activities (Calado et al., 2015). Different foods, fruits, beverages, herbal drugs are considered as good sources of flavonoids. Earlier studies have stated antimicrobial, anticancer, anti-inflammatory and anti-allergic activities due to effective scavenging of reactive oxygen species comprising of singlet oxygen and other free radicals (Montoro et al., 2005). The flavonoid content of the *S persica* fruit was estimated to be 317.48 ± 77.59 mg 100 g^-1^ DW (**Table 1**) which was found to be at par with the flavonoid content of plum (366 mg 100 g^-1^ DW) and was higher than that reported for blueberries (190.3 mg 100 g^-1^ DW) and other commercial fruits like apple, banana, peach, grapes etc. (García-Alonso et al., 2004; Marinova et al., 2005). A positive correlation of the flavonoid content with the high reducing power and antioxidant capacity of the plant was noted, suggesting the role of flavonoids in inferring these properties. Also, this leads to the consideration that there may be possible influence of anthocyanin pigments in this activity which provides dark red color to the ripe fruit. Thus, this fruit enriched with polyphenols and flavonoids, could be a potential source of antioxidants reducing risk of disease caused especially due to oxidative stress by increasing the overall antioxidant capacity of an organism.

### Mineral Ion Compositions of *S. persica* Fruit

The mineral ion compositions of the *S. persica* fruit are presented in **Table 2**. The potassium and calcium content was found high (1362.98 ± 81.50 and 1013.72 ± 48.60 mg 100 g^-1^ DW, respectively) which was higher than *S. oleoides* and *Dialium guineense* (Duhan et al., 1992; Marinova et al., 2005). Previous studies have suggested dietary intake of potassium has beneficial effect on coronary heart diseases by decreasing blood pressure and maintaining adequate Na^+^ and K^+^ ratio (Weaver, 2013). Calcium is a vital mineral nutrients for human diet and it is involved in many aspects of processes like muscle contraction, cell differentiation, neuronal activities and immune responses leading to program cell death (Pu et al., 2016). The calcium level in *S. persica* fruit was observed to be higher than in traditional fruits like banana, apple, orange, chiku, papaya, etc. and medicinal plant like *Caralluma tuberculata* (García-Alonso et al., 2004; Rathore, 2009), suggesting it to be a potential calcium source. The fruit extract was found to have significant Na^+^ (263.17 ± 28.32 mg 100 g^-1^ DW) concentration. The Na^+^ content was found to be higher than wild fruit *D. guineense*, *Chrysophyllum albidum, Irvingia gabonensis*, and *Cola millenii* (Olayiwola et al., 2013; Ayessou et al., 2014) revealing that *S. persica* is a good source of Na^+^ which is an imperative mineral for human health required for cellular homeostasis, fluid balance and important physiological processes (Farquhar et al., 2015). Magnesium is a central mineral for retention of vitamin D and calcium into the bones for maintaining bone structure and helps in prevention of osteoporosis and cardiovascular diseases (Chakraborti et al., 2002). Our result showed significant concentration of Mg^2+^ (143.33–159.36 mg 100 g^-1^ DW) much higher than that reported for other non-conventional fruits like *S. oleides, Prsopsis cineria, Capparis decidua*, *Ficus glomeratai,* and *D. guineense* but was comparable to *I. gabonensis* and less than that reported for *Spondias mombin* (Duhan et al., 1992; Olayiwola et al., 2013; Ayessou et al., 2014) suggesting the utilization of the fruit extract of *S. persica* in nutrient supplements as a source of minerals.

**Table 2:** Minerals profile of *S. persica* fruit.

Minerals	Minimum value	Maximum value	Average^a^
**Macrominerals (mg 100 g^-1^ DW)**			
Na	242.2	295.4	263.17 ± 28.3
K	1249.3	1436.6	1362.98 ± 81.5
Ca	956.5	1075.3	1013.72 ± 48.6
Mg	143.3	159.3	153.89 ± 7.4
P	97.1	169.7	143.03 ± 32.5
**Microminerals (μg 100 g^-1^ DW)**			
Fe	11578.9	16716.8	13790.92 ± 2157
Mn	772.8	775.6	773.87 ± 1.2
Zn	844.1	1046.7	959.85 ± 85.2

^a^*The values are mean ± SD of five independent replicates*.

The fruit of *S. persica* contained micronutrients Fe^2+^ (137.90 ± 21.57 μg 100 g^-1^ DW), Mn^2+^ (8.77 ± 1.79 μg 100 g^-1^ DW) and Zn^2+^ (8.95 ± 1.34 μg 100 g^-1^ DW) in considerable amount. Iron is vital for survival of an organism as it participate in various metabolic processes like respiration and DNA synthesis (Lieu et al., 2001). The Fe^2+^ content was found to be significantly comparable to *C. albidum*, *I. gabonensis*, *C. millenii* but was found lower than *S. oleides* and *D. guineense* (Duhan et al., 1992; Olayiwola et al., 2013). Manganese is an imperative mineral nutrient for human consumption but excessive intake may lead to neurodegenerative disorders (Avila et al., 2013). In accordance with this, the Mn^2+^content was found less in comparison to *S. oleiodes* and other wild fruits (Duhan et al., 1992) making it suitable for human intake. The trace element Zn^2+^ is required for optimal function of various physiological and biochemical processes. It also helps in healthy aging and mitotic division and DNA synthesis (Tudor et al., 2005). Zn^2+^ content was found to be comparable to that reported for *S. oleoides, Capparis decidua, Ziziphus jujube, I. gabonensis* (Duhan et al., 1992; Olayiwola et al., 2013) but was found lower than that reported for *D. guineense*, *C. albidum*, and *S. mombin* (Olayiwola et al., 2013; Ayessou et al., 2014).

### Antioxidant Capacity

**FIGURE 2:**
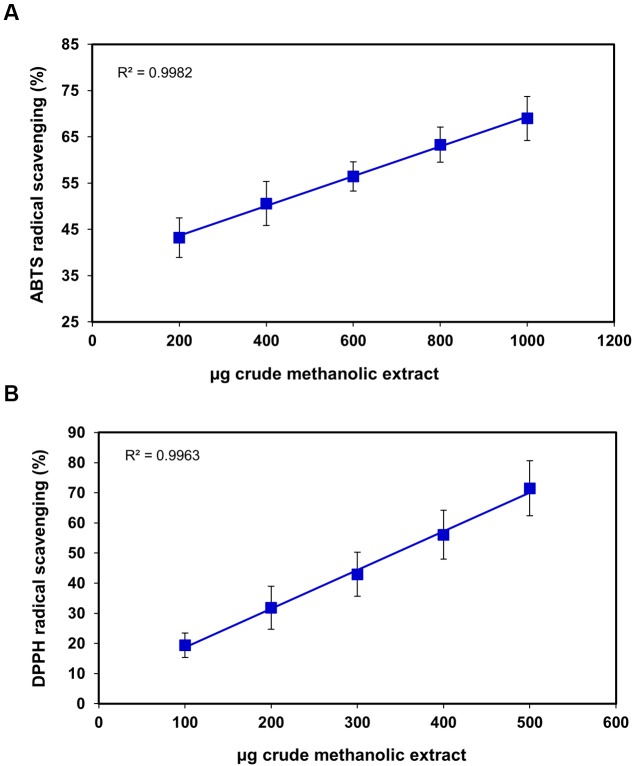
**Scavenging effects of *S. persica* fruit extracts on (A)** ABTS**^+^** radical and **(B)** DPPH radical.

**FIGURE 3:**
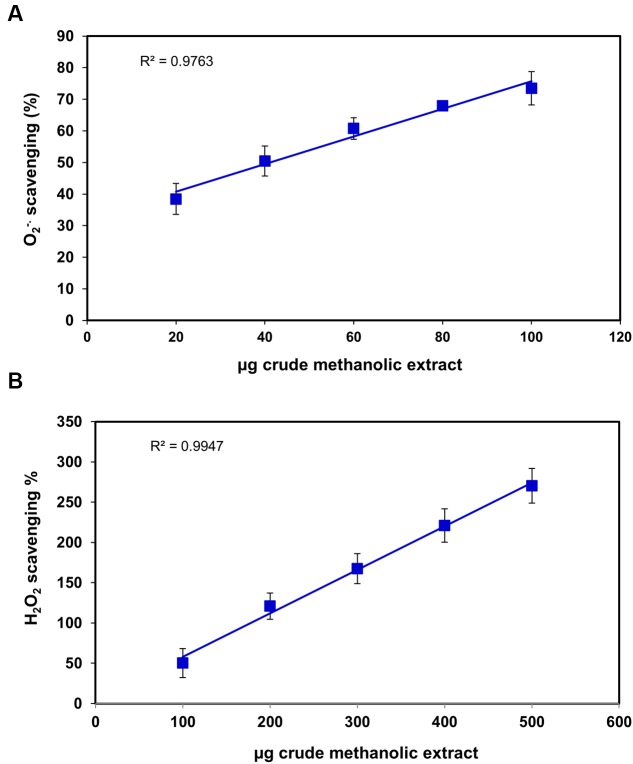
**Scavenging effects of *S. persica* fruit extracts on** **(A)** Superoxide radical and **(B)** Hydrogen peroxide.

**FIGURE 4:**
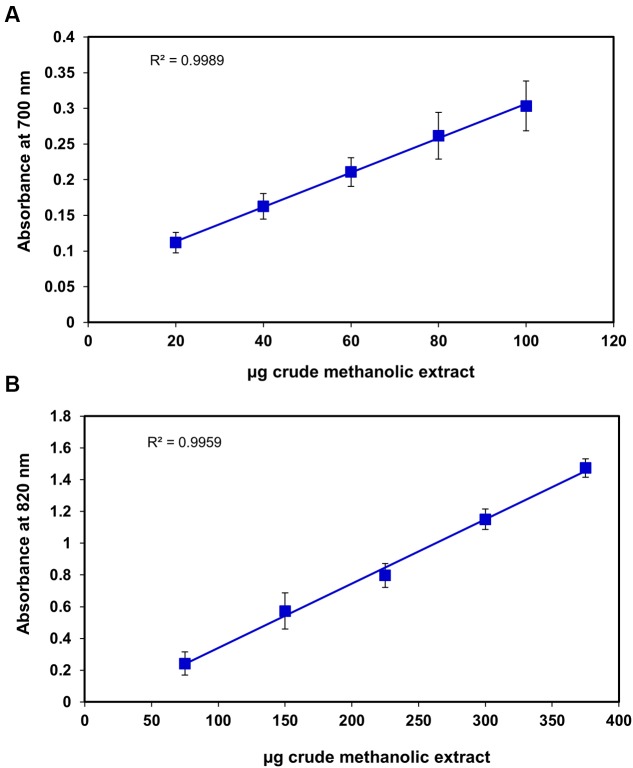
**Correlation between the antioxidant capacity and different concentrations of *S. persica* fruit extract as determined by** **(A)** Reducing power and **(B)** Phospho-molybdenum complex assay.

Nowadays, antioxidants in food are gaining high importance and are highlighted due to their pertinent role in preserving good health by prevention of diseases through scavenging the free radicals responsible for propagation of many and diseases like AIDS, cancer and neurodegenerative disorders. The current study composes the total antioxidant capacity of *S. persica* fruit extract presented in **Figures 2**–**4** assessed by ABTS, DPPH, hydrogen peroxide scavenging, superoxide scavenging, reducing power, and phosphomolybdenum complex forming assays. DPPH radical scavenging assay evaluates the ability to quench DPPH free radical (Prior et al., 2005). The IC_50_ for DPPH of the *S. persica* fruit was higher than guava, banana, star fruit and water apple (Lim et al., 2007) indicating low antioxidant potential in spite of its relatively high total phenolic content (IC_50_ = 307.06 ± 49.91 μg) (**Figure 2B**). The possible reason for that may be, first, it has been reported by Bondet et al., 1997 that low readings for reaction of DPPH for antioxidant activity may be due to reversible reaction of DPPH with phenols like eugenol and its derivatives. Another possible reason could be slow reaction rate between substrate molecules and DPPH (Huang et al., 2005). ABTS radical scavenging assay takes into account the reduction of ABTS ion to neutral colorless form for displaying the antioxidant capacity of the extract. The ABTS radical scavenging was found to be 365 ± 94.0 μg which was higher than that reported for guava (Thaipong et al., 2006) but was lower than chiku, strawberry, plum, star fruit (Leong and Shui, 2002) indicating higher antioxidant capacity than these commercial fruits.

Superoxide radical is regarded as a main source of ROS. It creates oxidative stress in the biological system by generating singlet oxygen and hydroxyl radicals and act as a weak oxidant. It arises due to electrolyte leakage from physiological processes and respiratory chain (Saeed et al., 2012). The hydrogen peroxide (H_2_O_2_) is a weak oxidizing agent which causes lipid peroxidation and damages DNA by production of hydroxyl radical (Hazra et al., 2008). The IC_50_ for superoxide and H_2_O_2_ radical scavenging was found to be 34.1 ± 1.2 μg and 85.70 ± 17.04 μg, respectively (**Figures 3A,B**), which was slightly higher than *Terminalia chebula, Terminalia belerica, Emblica officinalis,* and *Spondias pinnata* (Hazra et al., 2008, 2010) indicating significant free radical efficiency of *S. persica* fruit. The change in color from yellow to green caused by reduction of Fe^3+^ to Fe^2+^ demonstrates the reducing power of the extract, when measured at 700 nm (Saeed et al., 2012). The higher reducing power of *S. persica* fruit extract was shown by the increase in reducing power with increasing concentration. The correlation coefficient for this linear relationship was noted to be 0.998 (**Figure 4A**). The phosphomolymdenum assay measures the reduction of Mo (VI) to Mo (V) spectrophotometrically with absorbance maxima at 820nm for determining the antioxidant capacity. The antioxidant capacity was high as indicated by increase in absorbance with increasing concentration having correlation coefficient (*R*^2^) of 0.9932 (**Figure 4B**). The present study demonstrated that methanolic extract of *S. persica* fruit displayed the high antioxidant potential measured in terms of phosphomolybdate reduction. It has been reported that that the phosphomolybdate scavenging activity of many medicinal plants may be attributed to the flavonoid and related polyphenols present (Esmaeili et al., 2009). The higher redox potential of *S. persica* can be attributed to higher ascorbic acid and high phenolic content. Thus, owing to higher redox and antioxidant properties, *S. persica* fruit may serve as a source of antioxidants in pharmaceutical formulations and food supplements.

### Metabolite Identification by GCMS

**FIGURE 5:**
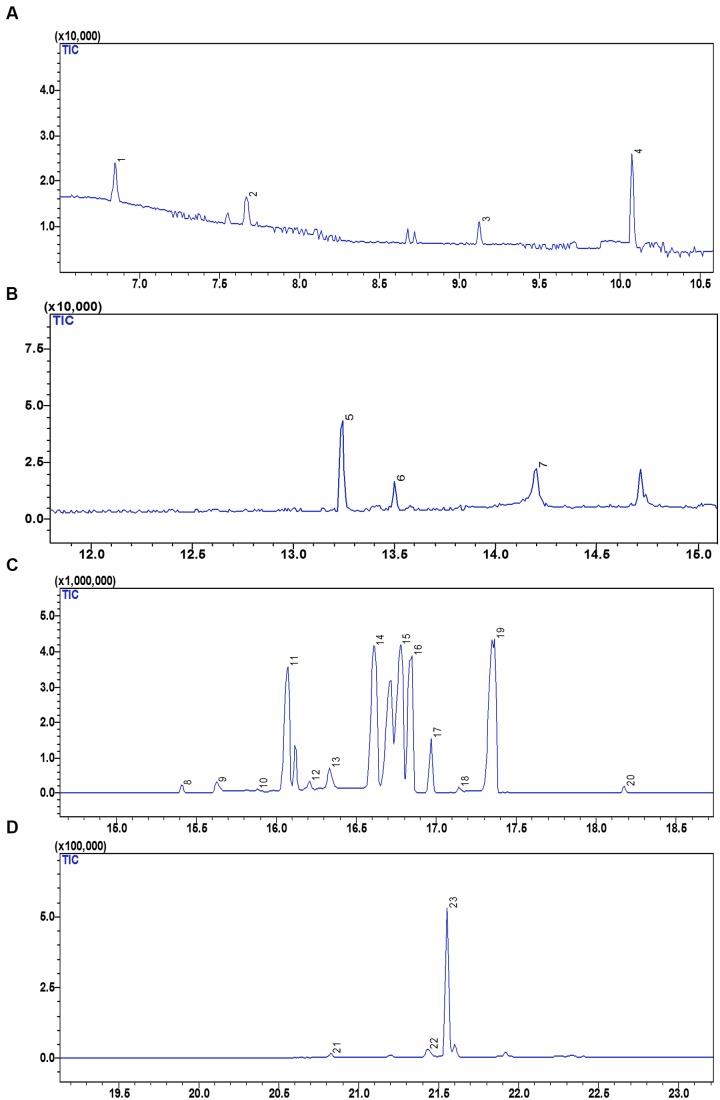
**GC-MS chromatogram representing the metabolic profile of *S. persica* fruit extract**. **(A)** GC-MS chromatogram between retention time (RT) of 7 to 11 min; **(B)** GC-MS chromatogram between RT of 13 to 15 min; **(C)** GC-MS chromatogram between RT of 14 to 18 min; **(D)** GC-MS chromatogram between RT of 18 to 23 min. The numbers mentioned above the peaks of the chromatogram refers to the sequence in which different metabolites were detected. Identities of peaks are given in **Table 3**.

The GC-MS analysis revealing the metabolite profile of the fruit extract of *S. persica* is represented in **Figure 5**. The peak identification, characteristics and quantification of metabolites are presented in **Table 3**. The GC-MS analysis of the fruit extract identified 22 metabolites having several pharmacological activities, antioxidant properties and nutraceutical potentials. The metabolites detected were grouped into organic acids, organic base, carboxylic acid, sugar alcohols and sugars. The identified metabolites are quantified using ribitol (peak 8, **Figure 5C**) as internal standard. The following headings discuss, in brief, the different nutritional and pharmacological functions of the identified metabolites from fruit extract of *S. persica*.

**Table 3:** Metabolites and their categories as analyzed in fruit extract of *S. persica* using GC-MS.

Categories of metabolites	Peak No.	Metabolites	Molecular ion [M-H]^-^ (m/z)	Quantity^a^
**Sugars (mg g^-1^ FW)**
Sugar	3	D-fructose	311	11.51 ± 0.1
	12	D-Fructopyranose	204	1.28 ± 0.1
	13	D-Allofuranose	319	0.10 ± 0.01
	14	D-Xylopyranose	307	1.34 ± 0.2
	15	D-Glucose	205	247.62 ± 8.7
	16	D-Mannose	206	26.30 ± 1.5
	17	D-Galactose	323	6.03 ± 1.3
	18	D-Xylose	218	0.92 ± 0.2
	19	Glucopyranose	217	42.90 ± 2.4
	23	D-Turanose	271	0.18 ± 0.01
**Others (μg g^-1^ FW)**
Organic acid	1	Lactic Acid	93	178.35 ± 37.5
	2	Oxalic acid	147	122.28 ± 8.8
	7	Benzoic acid	236	30.43 ± 0.9
Organic base	4	Guanidine	282	25.32 ± 0.3
Aromatic silica compound	5	Silane	341	33.80 ± 3.0
Nucleotide	21	Uridine	217	125.57 ± 2.9
Carboxylic acid	22	Butanedioic acid	288	140.38 ± 23.8
Hydroxy acid	9	Arabinonic acid	247	819.21 ± 29.9
	10	Mannonic acid	257	1145.09 ± 34.4
	11	Gluconic acid	217	4288.34 ± 390.6
Sugar alcohol	20	Myo-Inositol	320	1312.59 ± 228.4
	6	Glycerol	217	242.99 ± 42.0

^a^ The values are mean ± SD of six independent replicates.

#### Sugars

Sweet tasting, short chained carbohydrates are often called as sugars, which play important role in providing energy for human activities. Sugars are also used as the preservatives besides sweeting the food items. The fruit extract of *S. persica* as analyzed by GC-MS revealed 10 sugars, peak 3 (fructose having [M-H]^-^ at m/z 311.00), peak 12 (fructopyranose, [M-H]^-^ at m/z 204.00), peak 13 (allofuranose, [M–H]^-^ at m/z 319.00), peak 14 (xylose, [M–H]^-^ at m/z 307.00), peak 15 (glucose, [M–H]^-^ at m/z 205.00), peak 16 (mannose, [M–H]^-^ at m/z 206.00), peak 17 (galactose, [M–H]^-^ at m/z 323.00), peak 19 (glucopyranose, [M–H]^-^ at m/z 217.00), and peak 23 (turanose, [M–H]^-^ at m/z 271.00) (**Table 3**, **Figures 5B–D**). Glucose was quantified in highest amount (247.62 ± 8.77 mg g^-1^ FW) however; lower content was noted for allofuranose (0.10 ± 0.01 mg g^-1^ FW). Glucose is a vital source for production of proteins and lipids required for the normal metabolism of the human body. Besides this, it also acts as vitamin C precursor (Schauer et al., 2004). Fructose is referred as fruit sugar and it is a ketonic monosaccharide commonly found in plants. It is mainly used for sweetening food and beverages. Besides this, it is also found in cough suppressants, decongestant drops and liquids for children and adults, as well as nighttime medicines used for coughs and colds (Huttunen, 1971). As a diabetic sweetener, D-Xylose can be utilized in various industries including: beverages, pharmaceutical preparations, cosmetics, various foods and other industries. Besides this, D-Xylose is also utilized in moisturizers and beauty creams. Galactose and mannose functions as thickening agent, emulsifiers and food stabilizers in presence of oils or fats (Garti et al., 1997). The concentration of the identified sugars were found higher that that reported for olive fruits by Gomez-Gonzalez et al. (2010). Thus, the identified sugars add to the beneficial and nourishing value to the fruit of *S. persica*.

#### Sugar Alcohols

Sugar alcohol, also referred as polyols, are taken as good source of antioxidant and act as osmolyte for plants during stress condition (Raghavendra et al., 2013). The present study identified 2 polyols. Peak 20 and 6 were corresponds to myo-inositol and glycerol ([M–H]^-^ at m/z 320.00 and 217.00) (**Table 3**; **Figure 5**). The identified polyols have been previously reported to exhibit antimicrobial, anti-diabetic and chemoprotective activities (Raghavendra et al., 2013; Kim et al., 2015). Quantitative study of the identified metabolites indicated the presence of significant amounts of myo-inositol (1312.59 μg g^-1^ FW) and glycerol was (242.99 μg g^-1^ FW) suggesting chemoprotective and pharmacologically important nature of the fruit extract. Raghavendra et al. (2013) reported chemo-protective nature of myo-inositol suggesting its role for the treatment of depression by using it as a nutrient supplement. Similarly, antidiabetic property of glycerol has ben reported in stem extract of *Parkinsonia aculeate* (Kim et al., 2015). Owing to this property it is mostly used in pharmacological formulations. Besides this, the identified polyols are also reported to reduce dental caries having significant inhibitory effect on salivary lysozyme and enhances the activity of salivary peroxidase exhibiting good antibacterial activity (Raghavendra et al., 2013; Kim et al., 2015). Thus, the identified polyols content may be utilized in nutritional aspects.

#### Organic Acids

Organic acids are primary metabolites known to exhibit antimicrobial, antioxidant, and anti-tumorous effect and plays important role in plant metabolism as they are involved in several fundamental pathways (Kathirvel et al., 2014). Organic acids are mainly used in preparation of juices and beverages as food additives because they greatly influence the aroma, taste, and color of the food. The GCMS analysis of the fruit extract identified three organic acids (**Figure 5**) *viz.* lactic acid (peak 1, [M–H]^-^ at m/z 93.00), oxalic acid ([M–H]^-^ at m/z 147.00, peak 2) and benzoic acid ([M–H]^-^ at m/z 236.00, peak 7). Among the identified organic acids, the lactic acid was present in highest amount (178.35 μg g^-1^ FW) whereas the lowest content of benzoic acid was reported (30 μg g^-1^ FW). As compared to other metabolites oxalic acid was negligible signifying that as oxalic acid is considered anti-nutritional, its content should be lower in the edible part like fruit (Ayessou et al., 2014). Previous studies have reported contribution of benzoic acid and its derivatives to the flavor, aroma, and color of beer and some other beverages (Pereira et al., 2013). Besides this, the identified organic acids also used in food processing and preservation owing to their antimicrobial, antiviral, and high antioxidant properties (Sivasubramanian and Brindha, 2013).

#### Aromatic Silica Compound

Peak 5 [M–H]^-^ at m/z 341.00 was identified as silane (**Table 3**; **Figure 5B**). In harmony with our result, silane was also reported in *Euclayptus globolus* methanolic extract exhibiting antifungal activity (Al-Rahmah et al., 2011) and was also reported to contribute to antitumoral, antimicrobial activity of *Dryopteris cochleata* rhizome extract (Kathirvel et al., 2014). Silane has also been reported to have extensive uses in the industry as crosslinking agent, adhesion promoters, and surface modifiers (Plueddemann, 1991). Owing to the cross linking properties, silane finds enormous application as coupling agent in dentistry and also used for surface conditioning as adhesion promoter. The fruit of *S. persica* showed noteworthy amount of silane (33.80 μg g^-1^ FW) that can be utilized in dentistry industry.

### Amino Acid Profiling by HPLC

**FIGURE 6:**
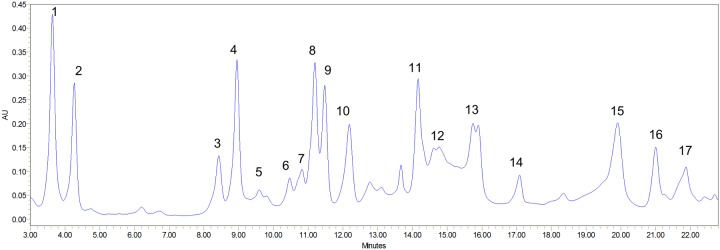
**Amino acid profiling of *S. persica* fruit extract as analyzed by HPLC**. The numbers mentioned above the peaks of the chromatogram refers to the sequence in which different amino acids were detected. Identities of peaks are given in **Table 4**.

Amino acids are nutrients that are imperative for enormous biosynthesis processes and are building blocks of protein that make up major content of human body. Apart from these basic functions they are also involved in providing other beneficial effects to human body which includes controlling of blood sugar level and building of muscles (Fürst and Stehle, 2004). Amino acids are majorly used as food additives in food industry and are also preferred as pharmaceutical and cosmetic industry due to their moisturizing effect (Banipal et al., 2016). The present study identified 17 amino acids which are listed in **Table 4** and representative chromatogram is shown in **Figure 6**. The identified amino acids can be calssified as essential, conditionally essential and non-essential amino acids and their role in food, pharmaceutical and cosmetic industry are discussed under these headings.

**Table 4:** Amino acids profile of *S. persica* fruit.

Peak No.	Amino acids (mg 100 g^-1^ DW)	Minimum value	Maximum value	Average^a^
1	Aspartic acid	40.7	53.6	48.57 ± 6.8
2	Glutamic acid	97.4	127.3	107.74 ± 17.0
3	Serine	42.6	54.3	49.70 ± 6.0
4	Glycine	57.4	76.9	66.65 ± 9.9
5	Histidine	126.4	154.6	140.58 ± 14.8
6	Arginine	44.2	32.2	37.34 ± 5.3
7	Threonine	63.0	77.1	69.82 ± 7.3
8	Alanine	31.4	38.0	34.69 ± 2.7
9	Proline	140.8	169.3	154.36 ± 14.3
10	Tyrosine	42.2	65.5	52.33 ± 10.1
11	Valine	126.6	134.6	129.62 ± 3.5
12	Methionine	129.1	183.5	156.98 ± 27.2
13	Cysteine	605.4	861.7	733.69 ± 107
14	Isoleucine	47.2	78.8	65.31 ± 16.3
15	Leucine	26.2	33.6	28.49 ± 3.4
16	Phenylalanine	80.7	100.7	93.57 ± 8.8
17	Lysine	23.7	31.5	26.78 ± 3.1

^a^*The values are mean ± SD of five independent replicates.‘*

#### Essential Amino Acids

Essential amino acid also referred as indispensable amino acid is an amino acid that cannot be synthesized by the organism and hence needed to be supplied from outside through diet. The nine amino acids which are considered essential include histidine, tryptophan, phenylalanine, leucine, isoleucine, lysine, valine, methionine, and threonine (Banipal et al., 2015). Essential amino acids are most importantly essential for usual functioning of human body Furthermore the synthesis of other amino acids and proteins also require essential amino acids (Banipal et al., 2016). They are also used to prevent fatigue and improve concentration. Leucine, isoleucine and valine are termed as branched chain amino acids and they are important for the regulation of carbohydrate metabolism, protein turnover and gene expression (Banipal et al., 2015). Peyrollier et al. (2000) reported key role of leucine as signaling molecule during synthesis of muscle proteins by controlling binding of 40S ribosomal subunit and mRNA. Additionally, the role of leucine in reversible phosphorylation of ribosomal protein S6 kinase has also been reported for the up-regulation of protein synthesis in skeletal muscle (Anthony et al., 2001). The amino acid profiling of *S. persica* fruit revealed of significant concentration of leucine (33.21 ± 10.23 mg 100 g^-1^ DW). L-Valine essentially play an important role as a central building block of various enzymes and is vital for supplying energy aiding to muscle cell repair and tissue recovery. Beside this, it also finds application in fermentation of beverages (Anthony et al., 2001). The valine content (129.62 ± 3.55 mg 100 g^-1^ DW) was found to be higher to *D. guineense* (Ayessou et al., 2014). The essential amino acid lysine is important for normal bone metabolism and has potential effect on factors influencing osteoporosis and healing of bones and was described to have positive effect on proliferation of osteoblast and synthetic activity and thus may help in prevention of many bone diseases (Torricelli et al., 2002). The essential amino acid concentration of the *S. persica* was slightly lower than that reported for wild fruits *Brachystegia eurycoma, Pipper guineense*, and *thonn* (Bolanle et al., 2014) but was found higher than that reported for *D. guineense* (Ayessou et al., 2014).

#### Conditionally Essential Amino Acids

Six amino acids comprising of glycine, cysteine, tyrosine, proline, glutamine, and arginine are referred as conditionally essential amino acids as their synthesis is limited to special pathological conditions such as severe catabolic distress and prematurity in infants. Cysteine is a sulfur containing amino acid synthesized from methionine. The dietary cysteine shows sparing effect on requirement of methionine (Fürst and Stehle, 2004). Cysteine has been earlier shown to have important role in protection against stressful conditions and an essential nutrient for immune response. Furthermore, cysteine and its derivative have been shown to modulate lymphocyte and macrophages activity. It also plays crucial roles in detoxification and protection of cells from free radicals and reactive oxygen species (Takahashi et al., 1997). The HPLC analysis of *S. persica* fruit estimated high concentration of cysteine (733.69 mg 100 g^-1^ DW) which was comparable to that reported for *Brachystegia eurycoma, Pipper guineense*, and *Thonn* (Bolanle et al., 2014). Glycine functions as key neurotransmitter in nervous system (Siegert et al., 2015). Besides this, it also helps in reducing blood sugar (Banipal et al., 2015). The HPLC analysis of *S. persica* fruit estimated 66.65 mg of glycine per 100 g DW. Proline is an α-amino acid widely used in food and pharmaceutical preparations. It is used as an osmoprotectant which protects membranes and proteins against damage by high concentration of inorganic ions (Banipal et al., 2016).

#### Non-essential Amino Acids

Non-essential or dispensable amino acids are those which can be synthesized by the organism. The present study identified five non-essential amino acids namely aspartic acid, alanine, glutamic acid, tyrosine and serine. Among these amino acids, glutamic acid was present in highest amount (140.48 ± 32.53 mg 100 g^-1^ DW), followed by aspartic acid and serine. Alanine was present in least concentration (36.43 mg 100 g^-1^ DW) (**Table 4**). In healthy individuals’ serine is synthesized from glycine and activated formaldehyde. Serine has been widely used in food pharmaceuticals and cosmetics industry. In food industry, it is mainly used in sports nutrition and besides this it is used in many cosmetic preparations as natural moisturizing agent (Banipal et al., 2016). Tyrosine is basically required for production of proteins and enzymes. It is produced from phenylalanine in the human body. It is used in dietary supplement for the treatment of depression and dementia (Siegert et al., 2015). Besides this, it is also utilized for drug delivery as an intercalating agent because of its structural resemblance with several drugs in pharmaceutical industry (Banipal et al., 2015). Thus, the identified amino acids in the fruit extract of *S. persica* showed their potential to be utilized in pharmaceutical industries.

## Conclusion

Nutrients, bioactive compounds, antioxidant activity, metabolite and amino acid profiling of *S. persica* fruit were analyzed. Depending upon the composition, the *S. persica* fruit has unique health benefits. The present investigation suggests that *S. persica* fruit is an enriched source of various bioactive constituents and has promising nutritional and antioxidant potential. This is the first complete study of the fruit of *S. persica* to investigate proximate composition, mineral ion contents, total metabolite, and amino acid profiling. This study has revealed that the fruit of *S. persica* is rich in essential mineral nutrients, proteins, soluble sugars, micronutrients, energy, antioxidant compounds, and other required constituents which can serve the malnutrition of the population group. In addition, the high antioxidant activity of the fruit extract of *S. persica* may be attributed for the presence of sugars, sugar alcohols, organic acids, amino acids, carotenoids and flavonoids. Furthermore, the high sugar content and strong antioxidant activity of the fruit might be exploited in the food industry as nutrient supplement and for increasing the shelf life of food products. These studies demonstrate the presence of high amount of mineral nutrients, ascorbic acid, secondary metabolites, and considerable amount of amino acids, which brings out the nutritional potential of the plant illuminating the likelihood of *S. persica* fruit for overcoming the malnutrition and as a source of non-conventional food and the future research would consider feasibility of preparation of confectionaries and other food products from the pulp of the fruit and large scale cultivation is recommended in salt effected coastal area on the basis of its high nutritional value and adaptability.

## Author Contributions

AK performed most of the experiments, prepared the manuscript, and analyzed the data. AKP designed and coordinated the experiments, interpreted the results, and improved the manuscript. JR and AP performed some of the experiments.

## Conflict of Interest Statement

The authors declare that the research was conducted in the absence of any commercial or financial relationships that could be construed as a potential conflict of interest.
